# Using KASP technique to screen *LRRK2* G2019S mutation in a large Tunisian cohort

**DOI:** 10.1186/s12881-017-0432-5

**Published:** 2017-07-06

**Authors:** Zied Landoulsi, Sawssan Benromdhan, Mouna Ben Djebara, Mariem Damak, Hamza Dallali, Rym Kefi, Sonia Abdelhak, Amina Gargouri-Berrechid, Chokri Mhiri, Riadh Gouider

**Affiliations:** 1 Department of Neurology, UR12SP21, Razi Hospital, 1 rue des Orangers, 2010 Tunis, Manouba Tunisia; 2 Laboratory of Biomedical Genomics and Oncogenetics, Institut Pasteur de Tunis, University of Tunis El Manar, BP 74, 13 Place Pasteur, 1002 Tunis, Tunisia; 3 Department of Neurology, Habib Bourguiba Hospital, 3029 Sfax, CP Tunisia; 4 Faculty of Medicine of Tunis, University of Tunis El Manar, Tunis, Tunisia; 5 National Institute of Applied Science and Technology, University of Carthage, Tunis, Tunisia

**Keywords:** Parkinson’s disease, LRRK2 gene, G2019S, KASP, genetic testing

## Abstract

**Background:**

In North African populations, G2019S mutation in *LRRK2* gene, encoding for the leucine-rich repeat kinase 2, is the most prevalent mutation linked to familial and sporadic Parkinson’s disease (PD). Early detection of G2019S by fast genetic testing is very important to guide PD’s diagnosis and support patients and their family caregivers for better management of their life according to disease’s evolution.

**Methods:**

In our study, a genetic PD’s diagnosis tool was developed for large scale genotyping using Kompetitive Allele Specific PCR (KASP) technology. We investigated *G2019S’s* frequency in 250 Tunisian PD patients and 218 controls.

**Results:**

We found that 33.6% of patients and 1.3% of controls were carriers. Demographic characteristics of patients with G2019S had no differences compared with non-carrier patients. Thereby, we could emphasize the implication of G2019S in PD without any distinctive demographic factors in the studied cohort. Sixty patients out of 250 were genotyped using Taqman assay and Sanger sequencing. The genotyping results were found to be concordant with KASP assay.

**Conclusions:**

The G2019S mutation frequency in our cohort was similar to that reported in previous studies. Comparing to Taqman assay and Sanger sequencing, KASP was shown to be a reliable, time and cost effective genotyping assay for routine G2019S screening in genetic testing laboratories.

## Background

In the last decade, genetic research has made tremendous progress in identifying the genetic etiology of Parkinson’s disease (PD). Mutations in *LRRK2* gene, encoding for the leucine-rich repeat kinase 2, were found to be the most frequent cause of late-onset autosomal dominant form of PD [1, 2]. The *LRRK2* gene, located on chromosome 12q12 with 51 exons, encodes a highly conserved 2527 amino-acid multi-domain protein having kinase and GTPase functions [3]. Among 80 missense variants in *LRRK2* gene, 7 mutations are associated with dominantly inherited forms of PD, and are clustered in functionally important regions [4]. The most prevalent *LRRK2* mutation is the c.6055 G > A in exon 41, which causes a glycine to serine substitution (G2019S) in the highly conserved activation loop of its MAP kinase domain [5]. The G2019S was shown to have a worldwide distribution [6]. Globally, this mutation occurs in 3 to 6% of familial PD and in 1 to 2% of sporadic PD [7]. However, its frequency varied greatly according to geographical or ethnic origin with the highest values seen in North African (37% of familial PD and 41% of sporadic PD) and Ashkenazi Jews (30% of familial PD and 13% of sporadic PD). Lower frequencies of this mutation were observed in sporadic and familial PD patients in North American (3 and 1%), European (4 and 1%) and Asian populations (0.1 and 0%) [6].

Although it is rarely found in healthy controls, genetic testing for *LRRK2* mutations is very important to support PD patients and their families. Thus, a fast and accurate genotyping method should be taken into account to screen the G2019S mutation. Early genotyping routine was commonly based on direct sequencing. This technique was then replaced by real-time polymerase chain reaction (PCR) based genotyping methods including TaqMan assay and high resolution melting technique. These methods are low-priced, less time-consuming and highly-sensitive. Recently, the Kompetitive Allele Specific PCR (KASP) genotyping assay has been successfully used to validate DNA polymorphisms. This uniplex, fluorescence-based genotyping technology, is based on allele-specific primer extension [8]. It offers the simplest and the most cost-effective way to determine precisely single-nucleotide polymorphism (SNP) genotypes.

In this work, we used KASP genotyping assay to screen the *LRRK2* G2019S mutation in a cohort of Tunisian PD patients. Through this technique, we aimed to develop a genetic diagnostic tool for large scale genotyping.

## Methods

### Patients and control subjects

A series of 250 Tunisian unrelated PD patients (96 familial and 154 apparently sporadic cases) were recruited at the Neurology Department, Razi Hospital (La Manouba, Tunisia). Patients were diagnosed as being affected if they satisfied the United Kingdom Parkinson’s disease Society (UKPDS) brain bank criteria, except the exclusion of family history of PD. In addition to PD patients, 218 Tunisian unrelated control subjects were recruited from different primary care clinics. They did not have symptoms or family history of neurological disorders.

### KASP genotyping assay

Genomic DNA was extracted from peripheral blood of subjects using the Salting-out method [9]. The 468 DNA samples, including 250 PD patients and 218 controls, were genotyped using the KASP assay.

The KASP assay mix required to determine SNP genotypes was purchased from LGC Genomics (Beverly, MA) using ‘KASP by Design’ method. The SNP of interest (rs34637584) and a 500 base-pairs (bp) sequence surrounding the SNP were annotated with known variants using ENSEMBL genome browser and were submitted to LGC Genomics website via their SNP submission template. The designed KASP assay mix comprised three primers (two allele-specific and one common reverse primer).

DNA samples were dispatched in 96-well plates. Two DNA controls were included in each reaction plate. The DNA controls were samples from one normal individual and a second individual heterozygous for the G2019S mutation selected from Whole Exome Sequencing (WES) data collected from the database of the Laboratory of Biomedical Genomics and Oncogenetics. No positive homozygous control was available. KASP reactions were done following the manufacturer’s protocol (LGC Genomics, Beverly, MA) using 50 ng of DNA in a 5 μl reaction volume. Reactions were carried-out in a 96-Well Light Cycler 480 II (Roche, CA) at the following conditions: 94 °C for 15 min, then 10 cycles of 94 °C for 20 s, 61 °C for 1 min, followed by 26 cycles of 94 °C for 20 s and 1 min at 55 °C. Following the PCR, fluorescence was detected using a single quantification cycle of 37 °C for 1 s after cooling at 37 °C for 1 min. Data were analyzed using the “Endpoint Genotyping” method of the Light Cycler 480 Software (release 1.5.0).

### TaqMan assay

Among the 250 PD patients, 60 were screened for *LRRK2* G2019S mutation by an allelic discrimination Taqman assay. According to the manufacture’s manual, the genotyping reaction was composed of TaqMan universal PCR master mix (Applied Biosystems Master Mix, catalogue number 4351379), TaqMan SNP genotyping reagent which included forward and reverse primers and SNP specific probe labeled with VIC dye conjugated to the 5′ end of the wild type allele probes and FAM dye conjugated to the 5′ end of the mutated allele probes. Allelic discrimination was automatically carried-out with PRISM 7000 sequence detection system (Applied Biosystems, CA).

### Sanger sequencing

Exon 41 of *LRRK2* gene was amplified by PCR from genomic DNA of 60 PD patients (already genotyped by Taqman and KASP assay) using two primers: 5′-TTTCCTGTGCATTTTCTGGCA-3′ and 5′-TGAGACAGACCTGATCACCTA-3′ under the following reaction conditions: denaturation at 95 °C for 5 min followed by 30 cycles of 95 °C for 30 s, 60 °C for 30 s and 72 °C for 30 s; and a final cycle of 7-min extension at 72 °C. PCR products were purified and sequenced using the Big-dye terminator Cycle Sequencing Kit v1.1 on an 3500xL Genetic Analyzer DNA sequencer (Applied Biosystems, CA). Sequences were analyzed using the Seqscape software (Applied Biosystems, CA).

### Statistical analyses

Statistical analyses were conducted using GraphPad Prism software version 5.03. The rs34637584 genotypes were examined for deviation from Hardy-Weinberg equilibrium (HWE) using Chi-square test. Statistical comparison of PD patients and controls with and without the G2019S mutation was performed where appropriate using Fisher’s exact test and F-test to compare variance.

## Results

Tunisian PD patients (250) and controls (218) were genotyped using the KASP assay. Our study revealed that the *LRRK2* G2019S mutation was present in 84 of 250 PD patients (33.6%) and in 3 of 218 controls (1.3%). Among the eighty-four G2019S carriers, 76 were heterozygous (30.4%) and 8 were homozygous (3.2%) for the mutation. No deviation from HWE was observed among PD patients (*P* > 0.05). All control G2019S carriers (3 individuals) were heterozygous. The frequency of the mutation among patients with familial cases (96 patients) was 37.5% (36 patients, 30 heterozygous and 6 homozygous) and 31% (48 patients, 46 heterozygous and 2 homozygous) in sporadic cases (154 patients).

In order to assess the accuracy of the KASP assay, genotyping results of 60 DNA samples of PD patients were verified through TaqMan assay and Sanger sequencing. Genotyping results were identical to those obtained by KASP.

Demographic characteristics of PD patients and controls were shown in Table 1. Both groups have equivalent sex ratios and similar mean age and age of onset. Demographic data of patients were stratified according to *LRRK2* G2019S (Table 2). The means of age of onset and gender distribution were not significantly different between carriers (homozygous and heterozygous individuals) and non-carriers. Furthermore, it seemed that the presence of G2019S mutation did not have an effect on the clinical picture of the sporadic nor on the familial form of PD. The mean age of onset was relatively similar in carriers (56.4 ± 13 for sporadic vs 55.8 ± 12.4 for familial, *p* = 0.76) and non-carriers (61.8 ± 10.8 for sporadic vs 55.8 ± 12.9 for familial, *p* = 0.12).

**Table 1:** Demographics of Patients and Control subjects

	ALL			LRRK2 G2019S carriers	
	Patients	Controls	*p*-value	Patients	Controls
N	250	218		84 (33.6%)	3 (1.3%)
Sex ratio (Men:Women)	1.2 (136:114)	1.1 (112:106)	0.51	1.1 (44:40)	2 (2:1)
Mean age (years, SD)	65.8 (10.8)	62.2 (10.5)	0.34	64.1 (11.5)	55.3 (7.5)
Median age (years, IQR)	66 (59–75)	61.5 (53–71)		64 (57–74)	55 (48–63)
Mean age at onset (years, SD)	58.5 (12.3)	–		56.1 (12.7)	–
Median age at onset (years, IQR)	59 (49–67)	–		57 (46–65)	–

**Table 2:** Demographics of PD patients with and without LRRK2 G2019S mutations

	LRRK2 G2019S Carriers			LRRK2 G2019S Non-carriers	*p*-value
	ALL	Homozygous	Heterozygous		
N	84	8	76	166	
Sex ratio (Men:Women)	1.1 (44:40)	1 (4:4)	1.1 (40:36)	1.2 (92:74)	0.68
Mean age at onset (years, SD)	56.1 (12.7)	56 (7.8)	56.2 (13.2)	59.6 (12)	0.44
Median age at onset (years, IQR)	57 (46–65)	57.5 (52–61)	57 (46–65)	60.5 (51–69)	

## Discussion

In the present study, we reported a large series of Tunisian patients in which the high frequency of the G2019S mutation seemed to be the cause of autosomal dominant PD. The mutation frequency among patients (33.6% of total, 37.5% of familial cases and 31% for sporadic cases) was in agreement with previously reported series of PD patients from Tunisia and North Africa. Frequency studies revealed a strikingly high value in North Africa (40.3% of familial and 33% of sporadic cases) [6]. Therefore, the G2019S mutation could be considered of clinical utility for the early molecular diagnosis and treatment of PD.

The mutation was also present in a 1.3% of control individuals. According to published reports, one of the highest frequencies of G2019S was found in North African controls (1.5%) [6]. Moreover, genetic screening in North African unrelated healthy subjects showed that the incidence of G2019S was 1.57% in 127 Tunisian controls [10]. However, the mean age of controls with G2019S mutation was lower than the general mean age at onset of PD. These individuals might be considered at risk carriers that could eventually develop PD within a decade or could probably be in the prodromal phase of PD.

Previous studies reported Tunisian families included in large cohorts of heterogeneous ethnic groups and G2019S frequencies were calculated for the whole North African population including Tunisia, Algeria and Morocco [7, 11, 12]. Specific data concerning the G2019S mutation in the Tunisian population were included only in three different reports summarized in Table 3. We noted that there was no overlap between our cohort and those described in previous studies. The first study reported a frequency of 42% in 91 Tunisian families [13] and a common ancestral founding haplotype was shared by these families and those from the United States, as well as from several European and Middle Eastern countries [14]. The second report was a case-control study performed on 238 patients with sporadic PD and 371 controls [15]. Later, clinical features of LRRK2 G2019S parkinsonism were studied in a 570 unrelated and 288 related patients including 220 sporadic (38%) and 126 familial cases (45%) who were carriers of the mutation [16]. In the same report, the mutation was also detected in 12 of 580 unrelated controls (3%).

**Table 3:** Summary of previous screening studies of the G2019S mutation in the Tunisian population

Reference	Type of study	Frequency in patients (%)	Frequency in controls (%)
Ishihara et al. 2007 [13]	Familial	38/91 (42%)	ND ^a^
Hulihan et al. 2008 [15]	Unrelated patients	72/238 (30%)	7/371(2%)
Trinh et al. 2014 [16]	Unrelated patients Related patients	220/570 (38%) 126/288 (45%)	12/580 (3%)
The present study	Unrelated patients	84/250 (33.6%)	3/218 (1.3%)

^a^ *ND* not defined

In our study, the age of onset was similar between *LRRK2* G2019S homozygous, heterozygous and non-carriers. Moreover, in these three groups, no significant differences of age of onset were shown when familial and sporadic forms were compared. These results were in accordance with previous Tunisian cohort studies [15, 16]. However, Ishihara et al. [13] reported that both homozygous and heterozygous G2019S individuals were older at onset than non-carrier patients. This difference could be explained by the familial nature of the study, including only related individuals (Table 3). In future studies, the screening strategy of the *LRRK2* G2019S mutation in the Tunisian population would focus on the recruitment of symptomatic individuals, regardless of age, gender or family history.

Through the development of whole genome sequencing by next-generation sequencing (NGS) technologies, a large number of polymorphic SNP linked to neurodegenerative disease were identified. The classic genotyping method using Sanger sequencing and PCR-RFLP were costly and labor-intense. Thus, a high-throughput and cost effective platform was required to carry out large-scale genotyping of these SNPs of interest.

The KASP genotyping system has been used widely and successfully over many years in the field of genetic improvement of animals and field crops [8]. In our work, we screened the rs34637584 in 250 PD patients using KASP genotyping assay. Sixty samples out of 250 were then screened by Taqman and Sanger sequencing in order to assess the reliability and the cost of methods used.

Unlike Sanger sequencing, both KASP and Taqman assays possessed mutual advantages, like low-labor, cost and high-tolerance of variability in DNA quantity and quality. The assays could be conducted for large numbers of samples within a short period of time without post-PCR handling [8, 17]. These properties are critical for a large-scale genetic testing. Moreover, KASP and Taqman genotyping PCR’s are suitable for use on a variety of real-time PCR instruments. However, the main drawback for these assays is their limited capacity to be used for SNP multiplexing.

Furthermore, KASP had higher advantage than Taqman. It did not require labelling of the SNP-specific primers which reduced the cost. Thus, KASP genotyping offered by different laboratory worldwide showed a significant cost saving option compared to common Taqman technologies. According to previous studies and some commercial genotyping service providers, the current KASP reaction costs five times cheaper than Taqman. Moreover, a study of SNP selection for the soybean genotyping [17] has shown that allele-specific assay cost 0.005 US$ for KASP compared to 0.238 US$ for Taqman.

In addition, KASP allows a greater flexibility for primer design which increased overall success rate and offerd an exceptionally accurate genotyping solution. Previous work has assessed the effectiveness of KASP genotyping technologies compared to Taqman or Sanger sequencing. According to an application note published by the University of Nottingham, the KASP technique was more efficient than TaqMan in terms of successful design of custom genotyping assays for a rare variant associated with Alzheimer’s disease [18].

The *LRRK2* G2019S mutation was screened in populations worldwide using classical targeted sequencing, High-resolution melting analysis and TaqMan genotyping assay. Ours was the first study using KASP genotyping to screen this recurrent mutation. KASP was used to genotype other SNP’s linked to PD, like the *LRRK2* variant rs1491942 and other SNPs in the *PARK16* locus in a Scandinavian population [19]. Besides, this technique was used to screen for variants in regulatory regions of *SNCA* associated with PD [20]. It was often required to confirm the association of rare variants with a particular phenotype like the rare R1205H and D620N mutations of *4G1* and *VSP* gene, respectively, in South African PD patients [21]. Moreover, KASP genotyping assay was recently used to screen SNPs in different neurological disorder like Alzheimer’s disease (rs75932628 of *TREM2* [22], rs1476679 of *ZCWPW1* gene [23] and both rs7412 and rs429358 for Apolipoprotein E genotyping [24]), amyotrophic lateral sclerosis [25], genetic generalized epilepsy (*VRK2* rs2947349 [26]) and polyglutamine diseases [27].

## Conclusions

By using KASP genotyping assay, we have shown that the G2019S mutation frequency in a PD Tunisian patient’s cohort was similar to that reported in previous studies. The KASP technique could be a reliable genotyping assay for routine mutation screening in genetic testing. The implementation of this diagnostic activity within hospitals in Tunisia and in other North African or emerging countries will pave the way for personalized medicine in these countries and could improve the management of PD patients and their families.

## Abbreviations

HWE: Hardy-Weinberg equilibrium
KASP: Kompetitive Allele Specific PCR
LRRK2: Leucine-rich repeat kinase 2
PCR: Polymerase chain reaction
PD: Parkinson’s disease
SNP: Single-nucleotide polymorphism
UKPDS: United Kingdom Parkinson’s disease Society
WES: Whole exome sequencing
